# Translational toxicology: a developmental focus for integrated research strategies

**DOI:** 10.1186/2050-6511-14-51

**Published:** 2013-09-30

**Authors:** Claude Hughes, Michael Waters, David Allen, Iyabo Obasanjo

**Affiliations:** 1 Quintiles, Inc, North Carolina State University; Wake Forest University; Duke University Medical Center, Morrisville, NC, USA; 2 Integrated Laboratory Systems, Inc, Research Triangle Park, Durham, NC, USA; 3 Advanced Leadership Fellow, Harvard University, Cambridge, MA, USA

**Keywords:** Translational toxicology, Environmental health, Biomarkers

## Abstract

**Background:**

Given that toxicology studies the potential adverse effects of environmental exposures on various forms of life and that clinical toxicology typically focuses on human health effects, what can and should the relatively new term of "translational toxicology" be taken to mean?

**Discussion:**

Our assertion is that the core concept of translational toxicology must incorporate existing principles of toxicology and epidemiology, but be driven by the aim of developing safe and effective interventions beyond simple reduction or avoidance of exposure to prevent, mitigate or reverse adverse human health effects of exposures.

The field of toxicology has now reached a point where advances in multiple areas of biomedical research and information technologies empower us to make fundamental transitions in directly impacting human health. Translational toxicology must encompass four action elements as follows:

1) Assessing human exposures in critical windows across the lifespan

2) Defining modes of action and relevance of data from animal models

3) Use of mathematical models to develop plausible predictions as the basis for

4) Protective and restorative human health interventions.

The discussion focuses on the critical window of *in-utero* development.

**Summary:**

Exposure assessment, basic toxicology and development of certain categories of mathematical models are not new areas of research; however overtly integrating these in order to conceive, assess and validate effective interventions to mitigate or reverse adverse effects of environmental exposures is our novel opportunity. This is what we should do in translational toxicology so that we have a portfolio of interventional options to improve human health that include both minimizing exposures and specific preventative/restorative/mitigative therapeutics.

## Background

In the current genomics era, gene-environment interactions provide the key to understanding health and disease. Human exposures to chemicals, physical agents and social factors are inevitable and determining what their subtle biological effect means to human disease is difficult. Absence of evidence of (effect or harm) is not evidence of absence (of effect or harm), particularly if adverse outcomes are ecologically apparent in environmental settings or in experimental animals following known exposures. To minimize adverse health outcomes, regulators, commercial entities, health care providers and the general public need to know what exposures pose a significant risk that merits intervention(s) to either reduce exposure or mitigate effects.

Multiple factors in the environment impact human development and subsequent health and disease. It has long been appreciated that vitamins, minerals and other essential nutrients play an important role in fetal growth and development. For example, the essential role of folate (folic acid) in pregnancy and human development is well known. But apart from a modest number of other well-known deficiencies, there is still limited understanding of the impact of micronutrient deficiencies or inadequacies on the fetus and subsequent long-term health outcomes [1]. Additionally, several socioeconomic and societal factors are known to have important effects. An unfolding example is the effect of maternal stress on pregnancy outcome which is still being elucidated.

The appreciation of maternal stress and other aspects of the social environment on the fetus has only recently gained some mechanistic explanation. Animal research suggesting transgenerational epigenetic programming of genes (maternal imprinting) has been seen as an attractive and plausible explanation. Recent reports in humans and nonhuman primates show that epigenetic effects may be sufficient explanations for such social effects. Radtke et al. [2] analyzed the methylation status of ten promoter sites for the glucocorticoid receptor (GR) gene in mothers and their children 10 to 19 years after birth. They paired those findings with the retrospective assessment of maternal exposure to intimate partner violence. Although the mother’s GR gene was not affected by that profile of intimate partner violence, the methylation status of the GR gene promoters of the adolescent children was influenced by their mother’s experience during pregnancy. If maternal exposure to intimate partner violence occurred during pregnancy, methylation of GR gene promoters was increased three-fold in the offspring [2]. In a broader profile of differential methylation of adult DNA, Borghol et al. [3] found that disadvantaged socioeconomic position in childhood was reflected in a change in the level of methylation for over 1200 gene promoters. While such a broad pattern of molecular effect does not explicitly link a disadvantaged background to the well-known adult mortality and morbidity risks, the findings certainly sustain the view that this is a biologically plausible mechanism. In nonhuman primates, Tunga et al. [4] used the social status (dominance rank) of female macaques to demonstrate that dominance rank imposed a widespread imprint on gene regulation such that gene expression data could (in reverse) predict social status with 80% accuracy. These remarkable correlations between methylation patterns and dominance rank strongly suggest that epigenetic molecular marks are a key response to social status effects during development. Taken together these human and nonhuman primate studies imply a distinct plausible association between social influence and individual physiology at a molecular level.

## Discussion

### Transformation of human Toxicology into a translational discipline: the translational gap

At present there is still a critical gap between laboratory toxicology research and testing (i.e., molecular, cellular and pathological outcomes in cell systems and animal models) and epidemiological and ecosystem studies, which examine exposed populations for disease. The current bipartite approach fails to make adequately overt the link between exposure(s) and clinical outcome(s). In fact, most such claims of cause-and-effect are no better than a tentative association of exposure and effect. So we are typically left with both, a) inadequate evidence of a plausible explicit risk due to an exposure, and b) inadequate evidence that there is no plausible explicit risk due to an exposure and therefore limited intervention possibilities. It is our objective to discuss current knowledge from research toxicology and epidemiological studies on the occurrence of adverse events triggered during critical life-span periods focusing on *in-utero* development and how to plausibly mitigate adverse health effects of these exposures particularly in terms of endocrine/metabolic, neurodevelopment and reproductive consequences across the ensuing lifespan.

#### *The public health imperative of endocrine and metabolic disorders*

If chemical exposures and stress influence the occurrence of endocrine mediated diseases, what is the potential public health impact? Golden [5] comprehensively reviewed the clinical and public health significance of 54 endocrine and metabolic disorders in the US, and the importance of any early in-life events that might impact subsequent endocrine or metabolic function associated with these disorders. Disorders with at least 5% prevalence among adults included diabetes mellitus, impaired fasting glucose, impaired glucose tolerance, obesity, metabolic syndrome, osteoporosis, osteopenia, mild-moderate hypovitaminosis D, erectile dysfunction, dyslipidemia, and thyroiditis. Even if the less common endocrinopathies are not considered, the high prevalence and incidence of common endocrine and metabolic disorders demonstrates the profound adverse impact of endocrine diseases on longevity, quality-of-life, public health and health care utilization.

**Endocrine and metabolic disorders: childhood obesity** As the world has moved from agrarian, to industrial, then information ages, the level of sedentary activities open to humans have increased exponentially, which has contributed to a global rise in obesity rates, affecting both low and high income countries, as well as creating wide variations within countries. In high income countries, the poor population is most affected while in low income countries, the most affected are the middle class [6]. This rise in obesity within populations forecasts an increased burden from several diseases, most notably cardiovascular diseases, diabetes, and cancers. Wang et al. [7] used a simulation model to project an increase of 65 million more obese adults in the US by 2030, coupled with in obesity-related medical costs reaching as high as $66 billion per year.

Obesity seems to activate disease by acting through sympathetic nervous activation. While the mechanisms responsible for initiating the pathway remain to be unequivocally elucidated, hyperinsulinemia, obstructive sleep apnea, increased circulating cytokines, stress and beta adrenergic receptor polymorphism are implicated in the causal pathway to diabetes and most of the other obesity related diseases [8]. For example, Chouchane et al. [9] found expression of TNF-alpha and stress protein (hsp70-2) associated with obesity and that these, and other endocrine disruptors, are most likely activated along the pathway leading to disease; thus they are potential targets for intervention. While diet and exercise is still an important therapy, the fact that these two simple interventions have not driven down the rising wave of obesity means that additional interventions are needed. Pharmacological interventions targeting the sympathetic nervous system, either directly or indirectly, may reduce disease outcomes and be useful along with improved nutrition and increased exercise.

As childhood obesity has steadily increased, legitimate public health concerns have been broadly raised. While there is great concern, there also is uncertainty as to the long-term health impact of this reality. On one hand, Birch et al. [10] suggest that the common notion that children will, "grow out of obesity," is not true and that critical environmental factors that affect children’s development and obesity risks in their early years should be modified in order to beneficially influence health and well-being throughout life. On the other hand, there remains a significant challenge to document whether childhood obesity is or is not an independent risk factor for adult metabolic syndrome, type II diabetes, or biomarkers of the same (blood lipid status, insulin levels, etc.) [11].

**Childhood obesity and *In-utero* environment** Over the last 20 years, childhood obesity has dramatically increased globally with accompanying increases in childhood illnesses associated with obesity. Childhood obesity leads to increased use of hospital services for several obesity-related conditions when compared to normal weight children [12]. More than a decade ago, Wang and Deitz [13] estimated that the cost associated with hospitalization for children due to obesity associated diseases at around 127 million for the two year period ranging from 1997 to 1999. Although obesity rates seem to have stabilized among females 2 to 19 years since 1999, it is still rising for males [14]. The associated health costs will continue to go up because the effects of obesity are cumulative causing more diseases and ill-health over time if not reversed. There is evidence that adult obesity is influenced by environmental factors and the same is probably true for childhood obesity, including prenatal and early postnatal environment. Salois [15] describes an association between the built environment (i.e., measures relating to food choice and physical activity) and prevalence of obesity in low income preschool children. There is an interaction between modern sedentary lifestyles, nutrition and *in utero* and early post-natal environments that influence the probability of childhood obesity. The *in utero* influences are likely the result of sympathetic nervous system activation in mothers leading to disruption of various endocrine pathways in the developing fetus. The *in utero* environment that triggers childhood obesity is not well understood. Various sympathomimetic compounds may be disrupting the *in-utero* environment and causing receptor changes that interact with the postnatal environment to produce phenotypic obesity. Such changes possibly could be detected in amniotic fluid. Possible sympathomimetic candidate compounds include environmental phenols, fungicides, pesticides and their metabolites, perfluorochemicals, phthalate metabolites and some metals.

**Childhood obesity and nutritional or metabolic imprinting** Epidemiological studies in human and animal experiments have shown that nutrition during fetal and neonatal life may lead to obesity and its related disorders in adulthood. There are no adequate models to reproduce human situations. For example, milk formula taken by infants during neonatal life may contain trace compounds of sympathomimetics that may produce subtle changes in receptor activation. Low birth weight is considered the result of an adverse *in utero* growth trajectory and is often associated with later metabolic diseases in adult age. The hypothalamic-pituitary-adrenal axis (HPAA) and growth hormone/insulin-like growth factor 1 (GH/IGF) axis may have a crucial role in the regulation induced by nutritional programming. The persistent alterations seem to be a consequence, at least in part, of elevated insulin levels during "critical periods" of pre- and early postnatal development. Likewise, leptin seems to play an important role in this complex system. New knowledge about these mechanisms suggests the development of new, rational, and effective preventive and/or therapeutic options before and/or after birth. Thus, early infancy may provide an opportunity for intervention aimed at reducing later disease risk [16].

#### *Effects of drugs and environmental chemicals on the reproductive system*

Clearly, drugs and environmental chemicals can adversely affect the reproductive system. Available data indicate that the consequences of exposure depend on the nature of the chemical, the dose of the chemical, its target, and the timing of exposure relative to critical windows in development of the reproductive system [17]. Toxicant exposures may affect critical events in reproductive development, ranging from early primordial germ cell determination to gonadal differentiation, gametogenesis, external genitalia, or signaling events regulating sexual behavior. Although there are differences between the human reproductive system and that of laboratory animal models, such models have been extremely useful in assessing risks for key human reproductive and developmental processes. The objectives for future studies should include the elucidation of the specific cellular and molecular targets of known toxicants; the design of a systematic approach to the identification of reproductive toxicants; and the development of sensitive, specific, and predictive animal models, minimally invasive surrogate markers, and/or *in vitro* tests to assess reproductive system function during embryonic, postnatal, and adult life. Integrated testing strategies will be required to account for the many mechanisms associated with reproductive development that occur *in vivo*.

Evidence from animal models and human studies indicates that many specific events can be perturbed by a variety of toxicants, with endocrine-mediated mechanisms being the more widely studied. Prioritized research needs include basic studies on the cellular-molecular and endocrine regulation using expanded animal studies on different classes of chemicals, comparing responses during development (prenatal and postnatal) with responses in adults; and, more extensive explorations regarding the reproductive biology and toxicology of puberty in humans [18].

**Folic acid and autism** Autism is a heterogeneous group of complex developmental disabilities that result from a number of possible etiologies. There is some debate about the increasing prevalence of autism and whether diagnosis of autism is increasing due to changing definitions and parents influencing the medical system for autism diagnosis for their children to access treatment for other ailments [19]. The 2010 prevalence estimate from meta-analyses of prevalence studies, 25 per 10,000, is up from the 1970 estimate of 1 per 10,000 [19]. Diagnostic and Statistical Manual of Mental Disorders (DSM) II was in use in 1970 and had different criteria for diagnosing autism from DSM IV-TR® in use in 2010. Diagnosis of autism is complicated because patients often have other co-morbidities such as seizures, GI problems, sleep disorders and attention deficit disorders. The causes of autism are still under active investigation with genetics, *in utero* exposure to antiepileptic drugs (valproate) [20] and GI bacteria being actively investigated as potential causes [21]. *In utero* hormonal disturbances are also indicted in causation of autism [22] and this is thought to explain the higher diagnosis of autism in males. High socio-economic status (SES) has consistently been found to be associated with autism but this could be due to under diagnoses in families with low SES [23]. Wang and Leslie [24] found that total expenditure for autism per child rose by 3.1% to $22,772 between 2000 and 2003 using Medicaid data from 42 states.

In summary, developmental exposure to some environmental chemicals appear to directly adversely affect neurodevelopment while other exposures that increase the incidence of metabolic conditions in mothers may be considered as an indirect cause as well. A recent publication indicates folic acid supplementation by mothers around conception and during pregnancy reduces risk of autism in children [25].

#### *Use of biomarkers as endpoints*

Numerous outcomes can be measured in human clinical trials. Particularly in therapeutic clinical trials (such as studies of pharmaceutical agents), investigators have the luxury of designing the studies to permit measurement of disease outcomes that would irrefutably demonstrate efficacy or lack of efficacy of the study drug. Hand-in-hand, alternative outcomes can be measured which are plausibly linked to the disease outcomes but may or may not be reliably associated with the ultimate outcome of interest. This conundrum has led to a classification of study outcomes (endpoints) that aids in the interpretation of study results. The National Institutes of Health have recommended definitions of outcome measures and these were nicely summarized by Grimes et al. [26] (See Table 1).

**Table 1:** **National Institutes of Health–recommended definitions of outcome measures**[26]

**Outcome**	**Definition**
Clinical end point	"A characteristic or variable that reflects how a patient feels, functions, or survives."
Surrogate end point	"A biomarker that is intended to substitute for a clinical end point. A surrogate end point is expected to predict clinical benefit (or harm or lack of benefit or harm) based on epidemiologic, therapeutic, pathophysiologic, or other scientific evidence."
Biological marker (biomarker)	"A characteristic that is objectively measured and evaluated as an indicator of normal biologic processes, pathogenic processes, or pharmacologic responses to a therapeutic intervention."

In our effort to develop a comprehensive approach to translational toxicology, we cannot anticipate relying on clinical endpoints that occur as a result of environmental exposures as the primary sensor for determining cause and effect relationships. Application of the precautionary principle has a strong ethical basis when the exposures of interest are unintended or unavoidable as occurs with numerous environmental health concerns. A study subject can give informed consent to take a study drug but individuals in an exposed population have given no such consent. Therefore in translational toxicology studies we can often expect to necessarily rely on surrogate endpoints or even biomarkers to drive decision-making and interventions.

The spectrum of potential biomarkers that may have utility in translational toxicology research is too broad to list. Beyond chemical and molecular markers such as clinical chemistry and hematology profiles, hormone levels [27], a multitude of imaging techniques (radiology images, ultrasound, CT, MRI, PET, etc.) can also be used. Histopathology remains a key component in assessing target tissue responses to exposures and new "-omics" technologies such as transcriptomics, proteomics and metabolomics as well as next generation sequencing for mutations present data sets that can serve as biomarker outcomes in various toxicologic investigations across the lifespan. For example, metabolomics have been applied to human embryonic stem cell test systems to begin identifying biomarkers of developmental toxicity [28]. Global metabolite profiling (untargeted metabolomics) allows the measurement of thousands of compounds in minimal amounts of biological material [29] and this technology can be applied broadly to any number of toxicity research inquires. These same "old" and "new" classes of biomarkers should be useful tools as we strive to assess the impact of interventions that may be implemented to mitigate the effects of prior or ongoing toxicant exposure.

Within the realm of genomics and RNA signaling, novel opportunities continue to arise. For example micro RNAs that are known to be key regulators of metabolism have been shown to circulate and therefore are available in accessible human specimens (blood) to quantify as either signaling molecules or disease markers [30]. Additionally, at the interface of molecular and cellular biomarkers, the recent report by Kim et al. [31] that the low levels of leukocyte mitochondrial DNA copy number are reduced in postmenopausal women with metabolic syndrome suggests that overt links between molecular markers, cellular markers, target tissue function and disease outcomes can be explicitly linked.

#### *Key developmental periods for interventions*

Key intervals in the lifespan that may be taken as Windows of Susceptibility (of adverse effects due to an exposure) are also likely to be the key intervals for intervention (Windows of Responsivity) to minimize, negate or reverse antecedent or ongoing exposure effects. Those key intervals of dynamic biological change during the lifespan that may be impacted by environmental exposures are:

• early development [pre-conception, *in utero*, neonatal, early childhood]

• peri-pubertal, and

• albeit in women only, the menopausal transition.

Effects of exposures can be one or more of the following:

• Create a new syndrome

• Recreate a known "old" syndrome

• Increase the prevalence or severity of some extant disease in an exposed population

• Exposure effects can interact with pre-existing unrecognized (such as common under-diagnosed) diseases, deficiencies, and other stressors to exacerbate later disease outcomes, either prevalence or severity.

There is also the possibility of unintended side-effects of interventions during the windows of susceptibility and this has contributed to reluctance to intervene during these periods.

**Early development** More than a decade ago, the U.S. Environmental Protection Agency convened a conference that addressed the concept of critical windows during development (preconception through puberty) when exposure to xenobiotics may have the greatest adverse impact on subsequent health. The potential impact of developmental exposures on reproductive health later in life [17,32] was judged to be especially vulnerable due to the multitude of potential cellular and molecular targets, the time dependency of essential developmental events and the numerous modes of toxicant action that can be anticipated. Key events occur within gametes before fertilization, across embryonic and fetal life, with additional maturational events occurring in the perinatal period, and at puberty. Complex genomic, hormonal, metabolic and growth signals are involved at all stages. The workshop group advised that future studies should include the elucidation of the specific cellular and molecular targets of known toxicants; the design of a systematic approach to the identification of reproductive toxicants; and the development of sensitive, specific, and predictive animal models, minimally invasive surrogate markers, or *in vitro* tests to assess reproductive system function during embryonic, postnatal, and adult life. Additionally, the panel emphasized that maternal exposures or stressors that needed to be further assessed included the following:

1. Psychosocial

2. Nutritional

3. Chemical exposures

4. Infectious disease exposures

5. Inter-current diseases (in mother)

6. Concomitant medications

7. Maternal body composition (obesity/fitness).

All of these themes persist today as important and inadequately defined areas of concern. As a single illustration, there is a strengthening view that several developmental exposures are causally associated with obesity later in life. Recent and ongoing epidemiological and animal studies have shown that up to 20 chemical endocrine disrupters are potential causes of obesity in children and adults if exposure occurs in early development [33]. Stress on the mother and nutrition of the mother have also been established to alter endocrine receptors and lead to obesity in children [9,16] from epidemiological and animal studies.

There is a need for further investigation to understand where effective interventions could be applied such as targeted nutritional guidance or active ways to reduce exposures of concern including possible prescription interventions for mother or child.

**Peri-pubertal** Given some recent studies showing that interventions in late adolescent/early pubertal children for risk of metabolic syndrome may have greater benefit than somewhat later interventions, the peri-pubertal interval may be a strong opportunity to show that environmental health impacts can be actively treated/mitigated/managed. A key concept is that the intrauterine fetal environment has effects that manifest in the late adolescent or pubertal interval.

***In utero* endocrinology and effects in adolescence** There is evidence in animals and humans that metabolic syndrome, insulin resistance, obesity etc. as well as female reproduction can be impacted by endogenous intrauterine endocrine or metabolic exposures. Since a number of animal studies had shown that exposure to intrauterine hyperglycemia appeared to increase risk factors for cardiovascular disease, Clausen et al. [34] studied the risk of overweight and metabolic syndrome in adult offspring of women who had diet-treated gestational diabetes or type I diabetes. These investigators assessed over 1000 women and were able to show that adult offspring of mothers with diet-treated gestational diabetes or type I diabetes have an increased risk of overweight and metabolic syndrome. These findings raise the question as to whether caloric restriction during gestation might reduce the risk of these adverse metabolic outcomes for the offspring. A recent study in rats [35], in which moderate caloric restriction of lactating rats was shown to protect from obesity development in the offspring as well as other related metabolic alterations, suggests that such a protective effect in humans is at least plausible.

While there has been extensive consideration of potential effects of exogenous sex hormone-like compounds on later reproductive potential of female offspring, less attention has been given to the potential effects of endogenous sex steroids during the intrauterine interval. Hart et al. [36] have addressed this question by assessing the impact of maternal circulating androgen levels during normal pregnancy on ovarian function, as determined by early follicular phase antimullerian hormone (AMH) levels, inhibin B levels, and antral follicle count (AFC) in 244 female offspring in adolescence. These investigators found that maternal circulating total testosterone levels at 18 weeks gestation were statistically significantly correlated with early follicular-phase circulating AMH levels in female adolescent offspring, but no other statistically significant correlations were determined among the maternal androgens and the markers of adolescent ovarian function at 18 or 34 weeks of gestation. Clearly such potential relationships between the intrauterine endocrine environment and the subsequent markers of reproductive competency in the peri-pubertal interval deserve further inquiry.

**Menopausal transition** The decade approximating age 40 to 50 years involves dynamic changes in reproductive endocrine functions and is temporally something of a "last chance" to preempt a number of risk profiles that influence diseases that become progressively more prominent in women after age 50–60. There is a moderate amount of attention now to endocrine, nutrition and lifestyle aspects of this life-stage, but virtually no attention is being given to the potential interaction of environmental chemical exposures with those themes. The menopausal transition results in changes in hormonal profile and changes in FSH, Inhibin B and AMH hormone have been noted; disruption of this intricate process may lead to abnormalities that could generate into disease [27,37].

### Documented human exposures from biomonitoring programs: what compounds/classes might be implicated?

#### *Chemical exposures of pregnant women: drugs, prescription and OTC*

The Food and Drug Administration (FDA) classifies drugs according to the degree of risk they pose for the fetus if they are used during pregnancy [38] (see Table 2).

**Table 2:** **Categories of risk for drugs during pregnancy **[38]

**Category**	**Description**
A	These drugs are the safest. Well-designed studies in people show no risks to the fetus.
B	Studies in animals show no risk to the fetus, and no well-designed studies in people have been done.
	or
	Studies in animals show a risk to the fetus, but well-designed studies in people do not.
C	No adequate studies in animals or people have been done.
D	Evidence shows a risk to the human fetus, but benefits of the drug may outweigh risks in certain situations. For example, the mother may have a life-threatening disorder or a serious disorder that cannot be treated with safer drugs.
X	Risk to the fetus has been proved to outweigh any possible benefit.

As a general matter, obstetrical care of pregnant women is precautionary and gravidas are discouraged from using prescription pharmaceuticals and over-the-counter (OTC) drugs to minimize exposure of the embryo or fetus to these classes of exogenous chemicals. Reality dictates that most women do in fact use one or more drugs during a pregnancy. More than 90% of pregnant women take prescription or nonprescription (over-the-counter) drugs or use social drugs (such as tobacco and alcohol) or illicit drugs at some time during pregnancy [38]. For the most part regarding this multitude of exposures, data are inadequate to support either our fearful assumption of fetal risk due to such exposures or to tacitly or explicitly endorse selective usage of drugs by gravidas. Given that common and presumptively safe drugs are also the ones frequently used by gravidas, some publications do document some of these *in utero* exposures and their potential fetal effects.

Acetaminophen (APAP) is one of the most frequently used drugs among pregnant women and is also the most common drug overdose in pregnancy [39]. Nonetheless, data regarding APAP overdose in pregnancy is limited and consists mainly of case reports or a small series of cases and there is no known threshold for occurrence of toxic symptoms. APAP crosses the placenta and high doses are known to be hepatotoxic. Hepatic metabolites may cause fetal hepatic necrosis. Because N-acetylcysteine (NAC) also crosses the placenta, some data suggest that the majority of morbidity and mortality from APAP overdose can be averted by initiation of NAC within the first 16 hours of ingestion and possibly even later. It appears that NAC does not itself pose any demonstrated risk to the fetus, so the standard-of-care would be to administer as early as possible after APAP overdosage in pregnancy.

Since the vast majority of usages of APAP are not overdosages, it has been important to determine if usage in proper dosages is or is not associated with an increased prevalence of congenital abnormalities and to determine a threshold for toxicity. Rebordosa et al. [40] assessed >88,000 pregnant women and their liveborn singletons from the Danish National Birth Cohort who had information on APAP use during the first trimester of pregnancy and 4.3% of children from the cohort were diagnosed with congenital abnormalities. Children exposed to APAP during the first trimester of pregnancy (n > 26,000) did not have an increased prevalence of congenital abnormalities compared with nonexposed children (n > 61,000).

In another study, Snijder et al. [41] examined the influence of maternal exposure to mild analgesics during pregnancy on the occurrence of cryptorchidism and hypospadia in >3000 offspring in the Netherlands Generation R [birth cohort] Study. Cryptorchidism and hypospadia were identified during routine screening assessments performed in child health care centers by trained physicians. The use of mild analgesics was assessed in three prenatal questionnaires in pregnancy, resulting in four periods of use, namely, periconception period, first 14 weeks of gestation, 14–22 weeks of gestation and 20–32 weeks of gestation. The prevalence was 2.1% for cryptorchidism and 0.7% for hypospadia. Use of mild analgesics in the second period of pregnancy (14–22 weeks) increased the risk of congenital cryptorchidism [with adjusted odds ratios (OR) of approximately 2] primarily due to the use of APAP. The results suggest that intrauterine exposure to mild analgesics, primarily APAP, during the period in pregnancy when male sexual differentiation takes place, increases the risk of cryptorchidism.

In summary, even for this extraordinarily common drug (APAP), we have data that makes advice to a pregnant woman rather equivocal. Namely, we do not think there is a risk of major congenital abnormalities if she were to use APAP during pregnancy but there is a doubling of risk of at least one congenital abnormality for male infants if she uses APAP in mid-gestation. If APAP is something of a "best case scenario" as regards having known developmental exposures and known clinical outcomes for the infants, the challenge to address all other relevant exposures (drugs, environmental and dietary chemicals, etc.) is indeed immense.

Arguably OTC drug usage could be deemed inessential, but some use of prescription drugs by pregnant women is clinically-compelling or truly unavoidable to assure her life and health. A prime example is the use of antiepileptic drugs (AEDs) [42]. For women with seizure disorders, many questions must be addressed even prior to conception. To avoid injury or death for the mother and the fetus, it may be impossible to stop or change the AED regimen during pregnancy. In some few cases an AED can be discontinued prior to conception, but this requires attentive and expert medical care. Most women with seizure disorders will require continued use of their AEDs before, during and after pregnancy. In practice, the aim is to prescribe the most effective AED while minimizing risks, and avoiding polytherapy and valproate if possible. Additionally, the dosing regimens will often require changes because drug metabolism of AEDs varies across pregnancy. Finally, the fetus faces risks due to potential effects of the AEDs, but also from the exposure to the concurrent maternal medical condition *per se* (her seizure disorder).

Another category of clinically-compelling prescription drug exposure *in utero* is antipsychotics. Antipsychotics are being used in behavioral medicine well beyond psychotic disorders to include depressive, bipolar, and anxiety disorders. As a result, fetal exposure to this class of compounds must be increasing in frequency, but any neurodevelopmental sequelae of fetal antipsychotic exposure is poorly understood. To examine whether intrauterine antipsychotic exposure is associated with deficits in neuromotor performance and habituation in 6-month-old infants, Johnson et al. [43] studied >300 maternal-infant dyads (N = 309) at 6 months postpartum with pregnancy exposure to antipsychotics (n = 22), antidepressants (n = 202), or no psychotropic agents (n = 85). Examiners masked to maternal-infant exposure status administered a standardized neuromotor examination (Infant Neurological International Battery [INFANIB]) that tests posture, tone, reflexes, and motor skills and a visual habituation paradigm using a neutral female face. Infants prenatally exposed to antipsychotics showed significantly lower INFANIB scores than those with antidepressant or no psychotropic exposure. These results indicate that 6-month-old infants who were exposed to antipsychotic drugs *in utero* had lower scores on a standard test of neuromotor performance suggesting that this was a neurodevelopmental effect of fetal antipsychotic exposure.

Finally, a recent study by Daw et al. [44] provides a broad and contemporary perspective of the extent of prescription drug use by pregnant women. The investigators assessed the frequency, timing, and type of medicines used before, during, and after pregnancy in a Canadian population. This retrospective cohort analysis used population-based health care data from all pregnancies ending in live births in hospitals in British Columbia from April 2001 to June 2006 (n = 163,082). Data from hospital records were linked to those in outpatient prescription-drug claims. Data from prescriptions filled from 6 months before pregnancy to 6 months postpartum were analyzed. Drugs were classified by therapeutic category and FDA pregnancy risk categories. Prescriptions were filled in 63.5% of pregnancies. At least 1 prescription for a category D or X medicine was filled in 7.8% of pregnancies (5.5% category D; 2.5% category X). The most frequently filled prescriptions for category D drugs were benzodiazepines and antidepressants. The most frequently filled prescriptions for category X drugs were oral contraceptives and ovulation stimulants filled in the first trimester. Remarkably, these data show that the majority of pregnant women in British Columbia received at least 1 prescription, and ~1 in 13 received a prescription for a drug categorized as D or X by the FDA.

This remarkable prevalence of maternal prescription drug use emphasizes the need for thorough pharmacovigilance and regulatory processes to assess potential fetal risks before, during and after drug approval and marketing. Additionally if a comprehensive translational approach is to be realized in human developmental toxicology, this category of fetal exposures must be included "in the mix" along with other environmental and dietary exposures.

Despite the widespread use of benzodiazepines during pregnancy and lactation, little information is available about their effect on the developing fetus and on nursing infants. Iqbal et al. [45] reviewed what is currently known about the effects of benzodiazepine therapy on the fetus and on nursing infants. Currently available information is insufficient to determine whether the potential benefits of benzodiazepines to the mother outweigh the risks to the fetus. High peak concentrations should be avoided by dividing the daily dosage into two or three doses. Minimizing the risks of benzodiazepine therapy among pregnant or lactating women involves using drugs that have established safety records at the lowest dosage for the shortest possible duration, avoiding use during the first trimester, and avoiding multidrug regimens.

#### *Chemical exposures of pregnant women: environmental and natural dietary chemicals*

Pregnant women and infants are exposed to multiple chemicals through the diet. These include naturally occurring dietary components such as phytoestrogens [46] as well as numerous synthetic compounds. The online database TEDX (The Endocrine Disruption Exchange 2013) [47] lists chemicals with the potential to affect the endocrine system. To date there are approximately 1,517 endocrine disruptors on the TEDX List.

Quantifying some of these dietary exposures may rely on survey instruments such as food frequency questionnaires as well as assays of biological specimens. Food frequency questionnaires (FFQ) have been adapted to provide a degree of quantification of soy consumption [48] by adult women. Since soy is the primary source of dietary estrogenic isoflavones in Western countries, the demonstrated correlation of data from the FFQ and plasma isoflavone concentrations indicate that either technique may provide adequate documentation of levels of exposure.

In recent years several different types of biological specimens from pregnant women have been assayed for environmental and natural dietary chemicals that are known to or suspected to have hormonal activity. Engel et al. [49] found detectable levels of three phytoestrogens (enterolactone, daidzein and genistein) and bisphenol A (BPA) in 21 amniotic fluid specimens that were collected before 20 weeks gestation. Samples were obtained by amniocentesis from women who were referred to the Mount Sinai Medical Center because of advanced maternal age. The relative concentration of the chemicals in amniotic fluid were enterolactone > daidzein > genistein > > BPA. Woodruff et al. [50] assessed exposure to environmental chemicals during fetal development by analyzing samples from pregnant women who were participants in the National Health and Nutritional Examination Survey (NHANES), a nationally representative sample of the U.S. population. The investigators analyzed data for 163 chemical analytes in 12 chemical classes for subsamples of 268 pregnant women from NHANES 2003–2004. The percentage of pregnant women with detectable levels of an individual chemical ranged from 0 to 100%. Certain polychlorinated biphenyls, organochlorine pesticides, perfluorinated compounds (PFCs), phenols, polybrominated diphenyl ethers (PBDEs), phthalates, polycyclic aromatic hydrocarbons, and perchlorate were detected in 99–100% of pregnant women. The median number of detected chemicals by chemical class ranged from 4 of 12 PFCs to 9 of 13 phthalates. Across chemical classes, median number ranged from 8 of 17 chemical analytes to 50 of 71 chemical analytes. In general, levels in pregnant women were similar to or lower than levels in nonpregnant women.

#### *Chemical exposures of pregnant women: herbs and supplements*

An additional category of potential chemical exposure of the human fetus that is neither drugs nor diet *per se* is the use of herbal products and dietary supplements. A recent national survey in the United States [51] demonstrates the widespread use of these biologically active products. These investigators assessed population and subpopulation changes in rates of herb and supplement use and rates of disclosure of herb and supplement use to conventional medical providers and compared data from the 2002 (n = 30,427) and 2007 (n = 22,657) Adult Complementary and Alternative Medicine File to the National Health Interview Survey (NHIS). The number of adults in the United States that ever used herbs or supplements was 55.1 million in 2007. The proportion of adults who reported use of herbs or supplements in the past 12 months was 17.9% in 2007. The proportion of respondents that disclosed herb or supplement use to their physician or another conventional medical professional was 45.4% in 2007. However, <1% of recent herb and supplement users disclosed this use to their pharmacist.

This study did not specifically address the question of usage of herbal products and dietary supplements by pregnant women and we are unaware of any reports documenting whether pregnant women are comparable to other adults in this regard.

#### *Chemical exposures of pregnant women: smoking, ethanol and drugs of abuse*

While animal studies have shown that early life exposure to cigarette smoke can have adverse effects on specific body systems such as the CNS [52], the adverse impact of tobacco (cigarette) smoking on the human fetus had been documented by a number of studies over the last three decades with most outcomes documented in terms of birthweights and/or other general measures of *in utero* growth. Wang et al. [53] reported on the relationship of birth outcomes to the timing and intensity of maternal active and passive smoking estimated both from self-reports and from cotinine concentration in maternal urine during early, middle, and late gestation. Their study included 740 white and Hispanic women in Boston, MA between 1986 and 1992. At each antenatal visit, information on maternal active and passive smoking was obtained by a detailed questionnaire, and by measurement of urine cotinine concentrations. Infant birth outcomes were obtained from hospital records. The percentage of mothers who ever smoked cigarettes during pregnancy was 55.5% for white and 10.2% for Hispanic women. A significant inverse exposure-response relationship between cotinine concentration in maternal urine and infant size (birthweight, length and head circumference) at birth was demonstrated. The relationship was less clear between maternal self-reported smoking status (both active and passive) and these fetal outcomes.

Additional studies have used various biological specimens for assessment of fetal exposure to tobacco smoking by measuring cotinine as the biomarker of exposure. Maternal salivary cotinine levels (SCLs) were compared to pregnancy outcomes among >700 pregnant women including 126 active smokers [54]. In linear regression analyses adjusting for sociodemographic and medical factors, SCLs of >20 ng/mL were associated with a reduction in birth weight of 88 g when SCLs were measured at baseline (P = .042) and 205 g when SCLs were measured immediately before delivery (P < .001). These investigators concluded that at any cutoff level, birth weight reduction was more significant for the same SCL measured in late pregnancy. Dietz et al. [55] analyzed data from the 1999–2006 National Health and Nutrition Examination Survey and estimated the percentage of 994 pregnant and 3,203 nonpregnant women 20–44 years of age who did not report smoking but had serum cotinine levels that exceeded the defined cut point for active smoking (nondisclosure). Active smoking was defined as self-reporting smoking or having a serum cotinine concentration that exceeded the cut point for active smoking. Overall, 13.0% of pregnant women and 29.7% of nonpregnant women were active smokers. Nondisclosure was higher among pregnant active smokers (22.9%) than among nonpregnant smokers (9.2%). The investigators concluded that studies and surveillance systems that rely on self-reported smoking status are subject to underestimation of smoking prevalence, especially among pregnant women, and underreporting may vary by demographic characteristics. In another study of fetal umbilical cord drug levels and maternal self-report [56] in >100 specimens, there was only fair agreement between self-reported smoking and cotinine levels as well as slight agreement with current drug use and positive drug levels. Compared with maternal self-report, sensitivity of cotinine levels was 27% and specificity was 98%. Sensitivity of positive cord illicit drug levels was 32% and specificity was 85%.A clear blunt summary of the adverse impact of tobacco smoking on women and their infants can be viewed on the Centers for Diseases Control website [57].

In terms of fetal exposure to ethanol, there is absolute certainty that pregnant women should not consume alcoholic beverages. This is a clear and certain case for fetal protection by avoidance of exposure and is an ongoing critically important public health message from the American College of Obstetrics and Gynecology [58]. A recent review and commentary by Tracy [59], succinctly and powerfully documents the history of fetal alcohol syndrome, the animal and human data indicating that there is no safe level of exposure and that obstetricians and other healthcare providers must be more assertive in identifying and counseling reproductive age women and especially those who are pregnant regarding fetal risk. This is a clear case where the toxicology, social norms and delivery of healthcare all make the course of appropriate action clear; the human fetus should not be exposed to ethanol.

Use of cocaine, marijuana, and other illicit drugs during pregnancy has been associated with a variety of adverse effects even though causality is not firmly proven. Effects include low birth weight, neurodevelopmental deficits and impairments affecting behavior, cognition, attention, language, and learning skills plus other behavioral problems in children exposed to cocaine, marijuana or heroin *in utero*. Methamphetamine exposure has been associated with fetal growth restriction, decreased arousal, and alterations in motor control in infants [60]. Neurotoxicity and psychopathology associated with methamphetamines and amphetamine exposure has been shown in both animal and epidemiologic studies, with more severe effects in women [61]. Given the high recreational use in young adults, the effects *in-utero* on the developing brains needs further investigation.

#### *Endocrine disruption as a functional category of toxicants*

The spectrum of possibly non-mutagenic but highly specific modes of action of chemical toxicants is implied by the molecular targets through which pharmaceuticals have been shown to act. The notion that some environmental and dietary compounds can act via highly specific signaling pathways to elicit reproductive and/or multifaceted developmental effects has led to appreciation of more subtle effects of exposures in animal models and raised suspicion that important developmental effects may be similarly occurring in humans that have gone unrecognized in the past. The full range of potential modes of action is captured in the "Guide to Receptors and Channels" [62]. This compilation of the major pharmacological targets is divided into seven sections: G protein-coupled receptors, ligand-gated ion channels, ion channels, catalytic receptors, nuclear receptors, transporters and enzymes. Would all of these actions be considered "endocrine disruption" or do we simply need to consider "developmental toxicology" to be the broader designation and limit "EDC" to those compounds that bind to or affect hormone signaling pathways *per se*?

Endocrine-disrupting chemicals (EDCs) can have effects at low doses that are not predicted by effects at higher doses [63]. Low-dose effects are those that occur within the range of human exposures or those observed at doses below the range used in traditional toxicological studies. These effects may depend upon nonmonotonic dose–response curves where a nonlinear relationship between dose and effect where the slope of the curve changes sign somewhere within the range of doses examined. It is noteworthy that nonmonotonic responses and low-dose effects are remarkably common in studies of natural hormones and EDCs. On this basis, Birnbaum [64] has urged collaborations between research scientists in academia, government, and industry to allow for development of more sophisticated study designs to facilitate regulatory decisions. Our core proposal is as follows:

In order to impact human health, the developmental toxicology concept of endocrine disruption must be "put to the test" in any instance where a scientifically valid health concern is raised in which:

a) human exposure(s) occur,

b) there is a plausible mode of action such that the exposure might elicit developmental effects,

c) human health outcomes are assessed and

d) both passive [reduce exposures] and active [nutritional, sociobehavioral or pharmaceutical interventions] health risk mitigation/intervention strategies can be developed for exposed individuals.

What evidence and modes of action link exposure to endocrine disruptors to endocrine or other human diseases? An essential missing link between endocrine disruptor testing and screening and epidemiology/population-based exposure assessments is individualized assessments of exposures and effects that are plausibly linked via known modes-of-action. Because metabolomic methods allow a broad look at multiple potentially perturbed metabolic pathways (and implictly different modes-of-action), and multiple biospecimens can be assayed [some of which are quite non-invasively obtained; urine], this may prove to be the preferred core analytical method for defining these individualized exposure-effect relationships.

### What modes of action are known or are plausible from animal, *in vitro*, and human clinical data?

Numerous biological processes and multiple levels of biological organization could be adversely impacted by exposures to exogenous chemicals and thereby affect fetal-neonatal development and measurable endpoints (see Table 3). These modes or sites of action include stem cell/progenitor cell numbers or lineages mutation, epigenetics, higher level effects on target tissue or body system(s) organization, stressors that affect CNS, inflammatory or immune pathways, etc.

**Table 3:** Developmental toxicology endpoints

**Endpoints**	**Methods of measurement**
General growth, morphology of body regions, organs and tissues	Classical teratology
Organization of organs & tissues	Microscopic veterinary or human pathology
Cellular composition of tissues	Relative composition of major cell types; histopathology, histochemistry, histomorphometry
Finer cellular and tissue profiles	Tissue content of stem cells and progenitor cells; inflammatory/migratory cell populations; extracellular matrix/ cell matrix interactions; assess cell cycle kinetics, critical cellular process (e.g., apoptosis and autophagy)
Molecular changes within cells	DNA, RNA, proteins (including processing, turnover, etc.), lipids and glycosylation; genomics, epigenomics, transcriptomics, microRNAs/siRNAs
"Accessible" or "translatable" (can be used in lab models and in humans) biomarkers	- Imaging (ultrasound, MRI, CT, etc.)
	- Functional assessments (behavior, treadmill, learning, cognition, renal function studies, lung function studies, grip strength, etc.)
	- Tissue biopsies, male and female gametes/follicular fluid; amniotic fluid; blood, urine
	- Routine chemistries, hematology,
	- Circulating cells (e.g., bone marrow stem cells, leukocytes, other stem/progenitor cells)
Biomolecules in blood plasma, urine including macromolecules, small molecules	- Specific assays for individual molecules
	- "Broad Net" approaches of proteomics, metabolomics, siRNAs, etc.
Integrative Physiology/Systems Biology	Multiple opportunities to link in vitro and animal models to humans and to use mathematical models to dynamically integrate multiple biological parameters

#### *Stem cell mediated effects*

One plausible target of toxic action in almost any target tissue during development is the stem or progenitor cell. Any exposure that might alter the population size or characteristics of stem or progenitor cells could have profound and lasting effects on the structural and functional integrity of any target tissue, organ or system. Additionally, since tools have been developed to study stem cells, the questions about potential actions of toxicants can now be investigated in relatively direct ways. In particular relationship to developmental effects of fetal exposures, stem cell populations from umbilical cord blood present a unique and well appreciated opportunity for study [65]. Umbilical cord blood contains at least three populations of stem cells, each with unique features and properties.

Hematopoietic stem and progenitor cells continuously egress out of the bone marrow (BM) and into the circulation under homeostatic conditions. This process is dramatically enhanced during stress situations and induced mobilization regimens [66]. Recent studies have clearly identified that hematopoietic stem cells (HSC) are part of a hierarchical organization defined by cells able to initiate long-term repopulation (LT-HSC) of injured BM followed by populations with transient repopulation ability [67]. As HSCs are able to supply blood cells for the lifespan of the individual, LT-HSCs undergo an aging process. The consequences of aging are an increase in the stem cell pool size, increased self-renewal and production of cells with expression of myeloid-specific genes and genes commonly expressed in myeloid leukemia. The age-related increased incidence of myeloproliferative disorders is therefore not surprising. Marrow cells circulate and migrate to a number of different organs. Previously assumed tissue and organ specificities appear to be less stringent and primitive cells residing in one organ may contribute to the structural and functional regeneration of other organs. It is intriguing to speculate that cells and cytokines used in appropriate concentrations may be able to repair damaged organs by using the defective organ as a scaffold, thus reducing the need for the transplantation of solid organs.

Evidence suggests haematopoietic stem cells (HSCs) can migrate to injured liver and influence tissue repair. However, Kavanaugh et al. [68] found that administered HSCs homed predominantly to lungs rather than liver, highlighting a potential therapeutic hurdle. Stem cells are potential targets of intervention as well as possibly targets of toxic agents.

#### *Mutagenesis and alteration of gene expression*

As a result of extensive collection of genome-wide transcriptional expression data from cultured human cells treated with bioactive small molecules, "The Connectivity Map (also known as cmap) has been assembled by use of simple pattern-matching algorithms [69]. This system enables the discovery of functional connections between drugs, genes and diseases through the transitory feature of common gene expression changes. At present the interface provides access to the current version (build 02) of Connectivity Map which contains more than 7,000 expression profiles representing 1,309 compounds. While not directly focused on toxicology or development, this broad and comprehensive approach toward characterizing the actions of chemicals on cells will surely be the type of valuable tool that will facilitate understanding the impact of toxicant exposures and potential mitigation strategies.

The types of advances being made are illustrated by recent work in hepatocellular carcinoma (HCC), the fifth most common cancer worldwide. HCC is more prevalent in men than women and recent genetic studies have revealed a causal role for androgen receptor (AR) in hepatocarcinogenesis, but the underlying molecular mechanism remains unclear. Feng et al. [70] used genome wide location and functional analyses to identify a critical mediator of AR signaling via cell cycle-related kinase (CCRK). Their results reveal a direct AR transcriptional target, CCRK, which promotes hepatocarcinogenesis through the upregulation of beta-catenin/TCF signaling.

#### *Epigenetic changes*

The field of toxicogenomics is undergoing remarkable expansion due to advances in genomic technologies as well as an expanding set of concepts about how environmental agents may impact gene regulation and heritability of traits. The epigenetic bases for heritable changes in gene expression (without accompanying alterations in the DNA sequence) are becoming more certain as mechanisms such as DNA methylation, histone modifications, non-coding RNAs, and changes in expression or activity of histone acetylases (HAT) and histone deacetylases (HDAC) in the regulation of gene expression patterns are elaborated [71]. It is becoming clear that epigenetic mechanisms play a critical role in normal development and differentiation and if disordered, may lead to diseases such as cancers. The growing body of evidence that environmental exposures especially in early development can induce epigenetic changes implies that these mechanisms will help explain toxicological effects of environmental exposures. Additionally, epigenetic changes are likely to explain a number of later-in-life effects of early-in-life exposures as well as effects in one or more subsequent generations.

The global prevalence of obesity is increasing across most ages in both sexes. Ng et al. [72] found that paternal high-fat-diet (HFD) exposure programs B-cell 'dysfunction’ in rat F1 female offspring. This report demonstrates that non-genetic, intergenerational transmission of the metabolic sequelae of a HFD from the father to his offspring occurs in mammals. Thus paternal as well as maternal exposures can bring about epigenetic alterations that influence the metabolic competency of their offspring.

Mathers et al. [73] summarized current knowledge of the impact of dietary, lifestyle, and environmental determinants of cancer risk and proposes that effects of these exposures might be mediated, at least in part, via epigenetic mechanisms. Evidence is presented to support the hypothesis that all recognized epigenetic marks (including DNA methylation, histone modification, and microRNA (miRNA) expression) are influenced by environmental exposures, including diet, tobacco, alcohol, physical activity, stress, environmental carcinogens, genetic factors, and infectious agents which play important roles in the etiology of cancer. Given the plasticity of epigenetic marks in response to cancer-related exposures, such epigenetic marks are attractive candidates for the development of surrogate endpoints which could be used in dietary or lifestyle intervention studies for cancer prevention. The authors argue that research should focus on identifying epigenetic marks which are (i) validated as biomarkers for the cancer under study; (ii) readily measured in easily accessible tissues (e.g., blood, buccal cells, or stool); and (iii) altered in response to dietary or lifestyle interventions for which there is convincing evidence for a relationship with cancer risk.

Transient environmental influences, such as perinatal nutritional stress, may induce deleterious metabolic symptoms that last for the entire life of individuals, implying that epigenetic modifications play an important role in this process. Jousse et al. [74] investigated, in mice, the consequences of maternal undernutrition during gestation and lactation on DNA methylation and expression of the leptin gene, which plays a major regulatory role in coordinating nutritional state with many aspects of mammalian biology. The investigators found that animals born to mothers fed a low-protein-diet (F1-LPD group) have a lower body weight/adiposity and exhibit a higher food intake than animals born to mothers fed a control diet (F1-CD group). These modifications persisted throughout life and were associated with lower levels of leptin mRNA and protein in starved F1-LPD mice, emphasizing that maternal protein-undernutrition affects the balance between food intake and energy expenditure in adults. Moreover, this nutritional stress resulted in the removal of methyl groups at CpGs located in the promoter of leptin, causing a permanent specific modification in the dynamics of the expression of leptin, which exhibits a stronger induction in the F1-LPD than in F1-CD mice in response to a meal. This study is an example of a molecular rationale linking transient environmental influences to permanent phenotypic consequences.

Histone modifications are thought to regulate chromatin structure, transcription and other nuclear processes. Histone methylation was originally believed to be an irreversible modification that could only be removed by histone eviction or by dilution during DNA replication. However, the isolation of two families of enzymes that can demethylate histones has changed this notion [75]. The biochemical activities of these histone demethylases towards specific Lys residues on histones, and in some cases non-histone substrates, have highlighted their importance in developmental control, cell-fate decisions and disease. Their ability to be regulated through protein-targeting complexes and post-translational modifications is also beginning to shed light on how they provide dynamic control during transcription.

O-GlcNAcylation, which is a nutrient-sensitive sugar modification, participates in the epigenetic regulation of gene expression. Thus, O-GlcNAc cycling may serve as a homeostatic mechanism linking nutrient availability to higher-order chromatin organization [76]. The wide range of physiological functions regulated by O-GlcNAc cycling suggests an unexplored nexus between epigenetic regulation in disease and nutrient availability.

To elucidate the role of age-related epigenetic changes in healthy ageing and potential longevity, Bell et al. [77] studied the association between whole-blood DNA methylation patterns in 172 female twins aged 32 to 80 with age and age-related phenotypes. Genome-wide analyses assessed differentially methylated regions (DMRs) for sixteen age-related phenotypes (ap-DMRs) and chronological age (a-DMRs). The majority of age-related changes in DNA methylation were not associated with phenotypic measures of healthy ageing in later life. Their results suggest that in a small set of genes DNA methylation may be a candidate mechanism of mediating not only environmental, but also genetic effects on age-related phenotypes.

### Translational research: biomarkers as a key component

Translatable biomarkers (e.g., true in animals and humans; such as hemoglobin or pulse rate) or biomarker equivalency/ pairings (organ weights or dimensions in animal models vis-à-vis CT, MRI or ultrasound imaging in humans) must be utilized but prudently. To advance understanding of whether exposures have effects that may be a risk, various molecular and imaging biomarkers have been developed, proposed and used in both laboratory and epidemiological settings. A critical shortcoming in this tactic is that a biomarker result is interpreted as a validated proxy for a clinical outcome. This assumption or interpretive leap is virtually always wrong. In human health research, the outcomes that truly matter are clinical endpoints (how a patient feels, functions or survives). Short of directly measuring clinical endpoints, surrogate endpoints can be measured if such surrogate endpoints have been validated. At a minimum for biomarkers to be plausibly interpreted as indicators of clinical endpoints, such biomarkers must be explicitly validated as predictors of surrogate endpoints. There are numerous such sets of endpoints and biomarkers that are used in medical practice with varying degrees of reliability. To illustrate, a few common examples would be as follows:

Clinical endpoint: sepsis

Surrogate endpoint: bacteremia

Biomarker: fever

Clinical endpoint: prostate cancer

Surrogate endpoint: microscopic pathology

Biomarker: prostate specific antigen (PSA)

Clinical endpoint: bone fractures

Surrogate endpoint: bone mineral density

Biomarker: osteocalcin (OC), trimeric carboxyterminal telopeptide (ICTP).

In environmental health research, we cannot have control over several key components in the "usual" human health research setting. Key characteristics of both the exposure (the agent, dose, duration, route, etc.) and the exposed subject (person’s age, sex, developmental lifestage, intercurrent illnesses, nutritional status, etc.) are not controlled by the investigator. So, for environmental health research where the aim is to assess possible human health effects of exogenous exposures, how can we approximate a thorough validation process of linking biomarker(s) to surrogate endpoint(s) to clinical endpoint(s)? We must transform the usual logic of "translational" health research into an approach that "works" to move toxicology research into actionable environmental health discoveries.

The aim of translational toxicology is to advance environmental health sciences in order to:

1. Transform Research & Development programs, especially endocrine disrupter programs, to link them overtly to disease outcomes;

2. Link target tissue/organ/system outcomes in animal models to animal biomarkers (mostly molecular) to human biomarkers to human target organ outcomes (pathology, imaging and functional)

3. Enhance predictive use of animal and human biomarker data sets by development of circumscribed computational (mathematical, statistical, knowledge-base, even simple schematic) model development.

### Timing of interventions for prevention or mitigation

We do need to consider that there may need to be sustained, continuing interventions for certain adverse effects/disease predispositions that are the result of environmental exposures. Nevertheless, key windows for interventions are likely to be the same as those in which adverse effects are elicited. Thus, in utero/neonatal/early childhood, adolescent/peri-pubertal and then menopausal transition in women may be particularly responsive since organizational/re-organizational processes are biologically dynamic in those intervals.

What are the prospects for screening pregnant women for exposures of concern and then implementation of mitigation strategies? Perhaps the current compliance of obstetrical practices in terms of testing in prenatal care is a guide. Siddique et al. [78] examined whether the frequency of four screening tests during prenatal care conforms to evidence of effectiveness. The investigators estimated rates of urine culture, anemia screening, oral glucose tolerance test (OGTT), and urinalysis during prenatal care by use of national probability samples of office visits to physicians (National Ambulatory Medical Care Survey) and to hospital outpatient departments (National Hospital Ambulatory Medical Care Survey) from 2001 to 2006. These data were compared to recommendations from the U.S. Preventive Services Task Force (USPSTF). On average, women received a urine culture in less than half of pregnancies. Women received just over one anemia screening on average per pregnancy. From 2001–2003, women received an average of 5.6 urinalyses per pregnancy; the average dropped to 4.3 urinalyses per pregnancy in 2004–2006. On average, women received just under one OGTT per pregnancy. Compared to younger white women, minorities and older women tend to get more anemia screenings, urine cultures, and OGTTs. Compared to USPSTF recommendations, too few women are receiving a urine culture during prenatal care. In contrast, women receive far too many urinalyses, but the rate appears to be falling. Anemia screening conforms closely to recommendations. The USPSTF does not recommend for or against universal diabetes screening using OGTT. Women appear to receive OGTT routinely.

### Obstetrical examples of interventions for fetal/neonatal/lifelong well-being

"Fetal therapy is broadly defined as any intervention administered to or via the mother with a primary indication to improve perinatal or long-term outcomes for the fetus or newborn." [79]. Due to concern about unknown consequences of fetal exposures, many leaders of public and private research entities, scientists, regulators and non-obstetrical physicians believe that no pharmaceutical interventions should be given to pregnant women for the purpose of enhancing lifelong well-being of the mother’s offspring. Beyond immunizations, care of maternal acute and chronic illnesses and specific obstetrical conditions (e.g., pre-eclampsia, gestational diabetes, etc.) including antenatal corticosteroids to enhance fetal lung maturity when premature delivery is anticipated, there are at least five different treatments that are commonly given to pregnant women where a primary aim is to effect fetal benefit that will obviously or likely manifest across the lifespan (see Table 4).

**Table 4:** Treatments commonly given to pregnant women for fetal benefit across the lifespan

**Disease/Indication**	**Treatment**	**Outcome**	**Selected references**
Neural tube defects	Folate	Reduction in risk of neural tube defects	American Academy of Pediatrics Committee on Genetics [80]
			U.S. Preventive Services Task Force [81]
Vision and cognitive function	Docosahexaenoic acid (DHA)	Support optimal visual and cognitive development	Koletzko et al. [82]
Smoking	Nicotine and/or N-acetylcysteine (NAC)	Smoking cessation and/or chemoprevention of DNA (and other) damage	Coleman et al. [83]
			De Flora et al. [84]
			Van Schooten et al. [85]
Maternal (and fetal)hypothyroidism	Thyroid hormone therapy	Avoid potential damage to neural development	Abalovich et al. [86]
			Rivkees & Mandel [87]
			Shields et al. [88]
			Kuppens et al. [89]
			Lazarus et al. [90]
Cerebral palsy associated with preterm delivery	Magnesium sulfate (MgSO4)	Reduction in risk of cerebral palsy/ motor disorders in childhood	Nelson & Grether [91]
			Rouse et al. [92]
			Conde-Agudelo & Romero [93]
			Rouse [94]
			Doyle et al. [95]

Two studies by Ibanez et al. [96,97] suggest that the peri-pubertal interval may be an important developmental window in which therapeutic interventions can be safely and effectively administered to mitigate the adverse health impact of earlier exposure to developmental stressors. Low-birthweight (LBW) girls with precocious pubarche (PP) are at risk for an early menarche (<12 years), an adult stature below target level, and PCOS. Hyperinsulinemic insulin resistance is thought to be a key factor. Therefore Ibanez et al. [96] studied the effects of early metformin treatment on menarche, height, and polycystic ovary syndrome (PCOS) markers as an open-label, randomized study in thirty-eight LBW-PP girls. At age 8 years, girls were randomly assigned to remain untreated or to receive metformin for 4 years; subsequently, both subgroups were followed without treatment until each girl was postmenarcheal. At last assessment, girls in each subgroup were on average 2 years beyond menarche; the mean growth velocity was below 2 cm/years. Age at menarche was 11.4 ± 0.1 years in untreated girls and 12.5 ± 0.2 years in metformin-treated girls; the latter girls were taller and much leaner (with less visceral and hepatic fat) and had more favorable levels of circulating insulin, androgens, and lipids. The conclusions were that early metformin therapy (age ~ 8–12 years) delayed menarche; augmented postmenarcheal height; reduced total, visceral, and hepatic adiposity; and curbed the endocrine-metabolic course of LBW-PP girls away from adolescent PCOS. In their second study, Ibanez et al. [97] compared the capacity of early vs. late metformin treatment to prevent adolescent PCOS. This was a randomized, open-label study over 7 yr in the thirty-eight LBW-PP girls who were followed up from the mean age 8 until age 15 yr. Early metformin was study yr 1–4; age 8–12 yr while late metformin was yr 6; age 13–14 yr. None of the girls dropped out of the study. At age 15 yr, early-metformin girls were taller (4 cm), were in a less proinflammatory state, and had less central fat due to reductions in visceral and hepatic fat. Hirsutism, androgen excess, oligomenorrhea, and PCOS were between 2- and 8-fold more prevalent in late- than early-treated girls. Abdominal adiposity was the first variable to diverge (at age 8–10 yr) between girls without vs. with PCOS at age 15 yr. The investigators conclude that in LBW-PP girls, early metformin therapy was found to prevent or delay the development of hirsutism, androgen excess, oligomenorrhea, and PCOS more effectively than late metformin. They suggest that the time window of late childhood and early puberty may be more critical for the development, and thus for the prevention, of adolescent PCOS than the first years beyond menarche.

### Concluding remarks

We need to broadly reorient perspectives to make toxicology and/or environmental health an active human health-promoting process. We cannot continue to rely on the existing dichotomy; on one hand, we have laboratory-based molecular, cellular and animal models data while on the other hand we have epidemiological and exposure assessment data. We are convinced that linking these types of data and scientific insights can be done and this is the crux of moving toxicology into an active translational research and interventional science.

Hormone signaling pathways may serve as mediators of reproductive and developmental effects and earliest indication of exposure. These early exposures may have later effects which may differ across the lifespan depending on other critical hormonal activities in the body.

Exposures are inevitable and the body either builds or has resilience or resistance to the adverse effects, or due to earlier exposures losses its ability to resist and succumbs to disease. The earlier exposures that cause the body to lose its ability to avoid disease in response to environmental factors needs to be evaluated.

Stem/progenitor cell populations must be a key component to recovery/resilience to insults and provide a capacity to recover/respond/heal either from the exposure of interest or to multiple future or concurrent insults later in development or across the lifespan.

Lab animals, ecological (wildlife) exposures, companion animals, and livestock can serve as bio-monitors and formal ways of getting observations from these populations into the knowledge base on human relevance need to be adopted.

A number of contemporary analytical techniques are available to detect and quantify exposures and effects. These methodological tools are widely called "biomarkers" and cover a rather wide range of techniques/technologies from DNA sequences of individual genes, to FACS of cell subpopulations, to 3-D reconstruction of tissues and organs from pathology slides, to whole body imaging with CT and/or fMRI, etc. Techniques such as "shotgun" proteomics and metabolomics offer further broad scope approaches for assessing responses of cells, tissues or organisms to external signals or stressors. Appropriate recognition of how biomarkers relate to disease outcomes in humans is critical to moving environmental toxicology from its current state to producing effective intervention strategies.

How do we best develop and gain insight by comparisons of critical windows in animal/cellular models to understand the human consequences for effects and the opportunities for protective interventions?

Because exposures occur to many individual compounds, classes of compounds and combinations (mixtures) of compounds and raise legitimate concern, translational links need to rely on various types of biomarkers that address two types of causal toxicopathologic pathways.

1. The one (or ones) that are known modes of action of the compound(s) such as most of the EPA Endocrine Disruptor Screening Program Tier 1 Screening Assays [98].

2. The possibility of unknown modes of action such as most of the EPA Endocrine Disruptor Screening Program Tier 2 Testing Assays [99] or use of metabolomics [99,100].

Number 1 is the "silver bullet" or "searching for the lost keys under the lamp-post" while number 2 is "casting the broad net upon the waters" or "searching in the dark." Both need to be done. Number 1 because it relies on using all that we know to trace a straight line between exposure, effect and prospects for mitigation; number 2 because chemicals (even those highly selected to have a very specific action via even a single molecular process, e.g., drugs) have actions in multiple molecular/cellular sites, and biological organisms and components are rife with pleiotropic and redundant systems. So, techniques that broadly ask what effects might result from exposures are essential for translational toxicology. The sorts of techniques that can "cast a broad net" to possibly let us see (previously) unknown modes of action include metabolomics, some forms of proteomics, assays of mutagenic and epigenetic effects, stem cell/progenitor cell/cell fate/cell sorting methods, histomorphometry and advanced microscopy techniques, circulating molecules (hormones, microRNAs, etc.), anatomical and functional morphology imaging methods. These approaches must be core components of the research and development strategy if translational environmental health is to advance and favorably impact public health.

## Summary

1. The pregnant woman and her fetus are exposed to many chemicals. These chemicals include environmental chemicals, dietary components, over-the-counter drugs, prescription drugs, substances of abuse (tobacco, alcohol and drugs of abuse) and herbal and related products.

2. Any of these may affect the fetus via many potential modes of action such as epigenetic, mutational, endocrine, stem cell, and a multitude of other signaling pathways (G protein receptors, ion channels, etc.).

3. We will depend upon use of biomarkers to assess actions of those compounds and to establish meaning for clinical outcomes. Readily measured biomarkers will have to be linked to surrogate outcomes which have to be representative of clinical outcomes.

4. In order to give substance to the term "translational", we must develop ethical and effective interventions to complement exposure reduction so that the adverse health effects of exposures can be prevented, reversed or mitigated.

## Abbreviations

a-DMRs: Chronological age; AEDs: Antiepileptic drugs; AFC: Antral follicle count; AMH: Antimullerian hormone; APAP: Acetaminophen (paracetamol); ap-DMRs: Age-related phenotypes; AR: Androgen receptor; BM: Bone marrow; BPA: Bisphenol A; CCRK: Cell cycle-related kinase; CDC: Centers for Disease Control; CNS: Central nervous system; CpGs: Cytosine-phosphate-guanine; CT: Computerized tomography; CXCR4+: C-X-C chemokine receptor type 4; DMRs: Differentially methylated regions; EDSTAC: Endocrine Disruptor Screening and Testing Advisory Committee; FACS: Fluorescence activated cell sorting; FDA: Food and Drug Administration; DHA: Docosahexaenoic acid; DNA: Deoxyribonucleic acid; DSM: Diagnostic and Statistical Manual of Mental Disorders; EDCs: Endocrine-disrupting chemicals; ETS: environmental tobacco smoke; F1-CD: Control diet; F1-LPD: Low-protein-diet; FFQ: Food frequency questionnaire; fMRI: functional magnetic resonance imaging; GH/IGH: Growth Hormone/Insulin-like growth factor 1; GR: Glucocorticoid receptor; HAT: Histone acetylases; HCC: Hepatocellular carcinoma; HDAC: Histone deacetylases; HFD: High-fat-diet; HPAA: Hypothalamic-pituitary-adrenal axis; HSC: Hematopoietic stem cells; ICTP: Trimeric carboxyterminal telopeptide; INFANIB: Infant Neurological International Battery; IQ: Intelligence quotient; LBW: Low-birthweight; LT-HSC: Long-term repopulation of hematopoietic stem cells; Lys: Lysine; MgSO4: Magnesium sulfate; miRNA: Micro-ribonucleic acid; MRI: Magnetic resonance imaging; NAC: N-acetylcysteine; NHANES: National Health and Nutritional Examination Survey; NHIS: National Health Interview Survey; OC: Osteocalcin; O-GlcNAc: O-GlcNAcylation; OGTT: Oral glucose tolerance test; OR: Odds ratio; OTC: Over the counter; PBDEs: Polybrominated diphenyl ethers; PCOS: Polycystic ovary syndrome; PET: Positron emission tomography; PFCs: Perfluorinated compounds; PP: Precocious pubarche; PSA: Prostate specific antigen; RNA: Ribonucleic acid; RT-PCR: Reverse transcriptase polymerase chain reaction; SCLs: Salivary cotinine levels; SDF-1: Stromal-cell-derived-factor-1; SES: Socio-economic status; siRNAs: Short inhibitory ribonucleic acids; TCF: T-cell factor; TCSCs: Tissue-committed stem cells; TEDX: The Endocrine Disruption Exchange; USPSTF: U.S. Preventive Services Task Force; VCAM-1: Vascular cell adhesion protein 1.

## Competing interests

All authors [C.H., M.W., D.A., and I.O.] declare that they have no competing interests. Quintiles, Inc. [C.H.] is a pharmaceutical services company that conducts pharmaceutical and biotechnology research and product development for numerous sponsors but does not own pharmaceutical or biotechnology products. Integrated Laboratory Systems, Inc. [M.W. and D.A.] is a research and testing services company that does not own pharmaceutical or biotechnology products.

## Authors’ contributions

All authors researched and wrote the debate. All authors provided critical reviews and revisions of the manuscript. All authors read and approved the final manuscript.

## Authors’ information

CH – MD, PhD: Executive Director, Cardiovascular and Metabolic Unit, Quintiles, Inc., Morrisville, NC, USA; Department of Obstetrics & Gynecology, Duke University Medical Center; Department of Mathematics, North Carolina State University; Department of Pathology/Comparative Medicine, Wake Forest University

MW –PhD: Chief Scientist, Integrated laboratory Systems, Inc., Research Triangle Park, NC, USA.

DA – PhD: Chief of Staff, Integrated laboratory Systems, Inc., Research Triangle Park, NC, USA.

IO – DVM, PhD: Advanced Leadership Fellow, Harvard University, Cambridge, MA, USA.

## Pre-publication history

The pre-publication history for this paper can be accessed here:

http://www.biomedcentral.com/2050-6511/14/51/prepub
